# An antagonistic monoclonal anti–Plexin-B1 antibody exerts therapeutic effects in mouse models of postmenopausal osteoporosis and multiple sclerosis

**DOI:** 10.1016/j.jbc.2022.102265

**Published:** 2022-07-15

**Authors:** Melanie Vogler, Arkadiusz Oleksy, Sabrina Schulze, Marina Fedorova, Baktybek Kojonazarov, Sharandip Nijjar, Seema Patel, Sian Jossi, Kovilen Sawmynaden, Maud Henry, Richard Brown, David Matthews, Stefan Offermanns, Thomas Worzfeld

**Affiliations:** 1 Department of Pharmacology, Max-Planck-Institute for Heart and Lung Research, Bad Nauheim, Germany; 2 LOEWE Center for Translational Medicine and Pharmacology, Frankfurt, Germany; 3 LifeArc, Accelerator Building, Open Innovation Campus, Stevenage, United Kingdom; 4 Institute for Lung Health (ILH), University Hospital Giessen and Marburg, Medical Clinic II, Giessen, Germany; 5 Faculty of Medicine, University of Frankfurt, Frankfurt, Germany; 6 Institute of Pharmacology, University of Marburg, Marburg, Germany

**Keywords:** antibody, monoclonal antibody, multiple sclerosis, experimental autoimmune encephalomyelitis, osteoporosis, plexin, semaphorin, plexin-B1, Sema4D, CD100, EAE, experimental autoimmune encephalomyelitis, MS, multiple sclerosis, SA, streptavidin, scFv, single-chain variable fragment, Sema4D, semaphorin 4D, SPR, surface plasmon resonance

## Abstract

Osteoporosis and multiple sclerosis are highly prevalent diseases with limited treatment options. In light of these unmet medical needs, novel therapeutic approaches are urgently sought. Previously, the activation of the transmembrane receptor Plexin-B1 by its ligand semaphorin 4D (Sema4D) has been shown to suppress bone formation and promote neuroinflammation in mice. However, it is unclear whether inhibition of this receptor–ligand interaction by an anti–Plexin-B1 antibody could represent a viable strategy against diseases related to these processes. Here, we raised and systematically characterized a monoclonal antibody directed against the extracellular domain of human Plexin-B1, which specifically blocks the binding of Sema4D to Plexin-B1. *In vitro*, we show that this antibody inhibits the suppressive effects of Sema4D on human osteoblast differentiation and mineralization. To test the therapeutic potential of the antibody *in vivo*, we generated a humanized mouse line, which expresses transgenic human Plexin-B1 instead of endogenous murine Plexin-B1. Employing these mice, we demonstrate that the anti–Plexin-B1 antibody exhibits beneficial effects in mouse models of postmenopausal osteoporosis and multiple sclerosis *in vivo*. In summary, our data identify an anti–Plexin-B1 antibody as a potential therapeutic agent for the treatment of osteoporosis and multiple sclerosis.

Plexins comprise a family of transmembrane receptors for semaphorins, which are membrane-bound or diffusible factors that regulate key cellular functions (1, 2, 3). The semaphorin-plexin system is of critical importance for cell–cell communication in multiple biological contexts, including the bone and immune system (4, 5, 6). Due to its central role in pathophysiological processes, the semaphorin-plexin system represents a promising drug target (3). In mammals, based on homologies, plexins are divided into four subfamilies (A-D), and semaphorins are grouped into five classes (7). The interaction of plexins with semaphorins is mediated by a highly conserved seven-blade β-propeller domain, the “sema domain” (8, 9, 10).

Osteoporosis is a highly prevalent disease, which is characterized by low bone mass and represents the most common cause for bone fractures among the elderly (11). A central pathogenetic factor in osteoporosis are the imbalanced activities of the bone-forming cells, osteoblasts, and the bone-resorbing cells, osteoclasts (12). The mainstay of currently used therapies for osteoporosis are antiresorptive agents that inhibit the activity of osteoclasts (11). Those include bisphosphonates such as alendronate, estrogens, selective estrogen-receptor modulators such as raloxifene, and the anti-RANKL antibody denosumab. However, adverse effects of these antiresorptive drugs, in particular of bisphosphonates, and the absence of clear evidence in support of their long-term efficacy raise concerns about their use (13). So far, only a few anabolic therapies are available, which, in contrast to antiresorptive agents, promote the activity of osteoblasts and increase bone formation. The caveat of these anabolic agents—which are parathyroid hormone and parathyroid hormone–related peptide analogs such as teriparatide and abaloparatide and the anti-sclerostin antibody, romosozumab—is that their anabolic effect is waning only after several months, which limits their clinical utility for long-term therapy (14). Moreover, teriparatide and abaloparatide cannot be administered for longer than 2 years due to a potential risk of osteosarcoma formation (14). Therefore, and in light of the increasing median age of the global population, new therapeutic strategies for the treatment of postmenopausal osteoporosis are of high medical importance. The plexin family member, Plexin-B1, is expressed by osteoblasts, while its high-affinity ligand, semaphorin 4D (Sema4D), localizes to osteoclasts (15). The binding of Sema4D to Plexin-B1 potently inhibits bone formation *via* activation of the small GTPase RhoA and suppression of insulin-like growth factor-1 signaling in osteoblasts (15). In line with this, mice lacking Sema4D or Plexin-B1 as well as mice expressing dominant-negative RhoA specifically in osteoblasts display significantly increased bone mass (15, 16).

Multiple sclerosis (MS) is the most prevalent chronic inflammatory disease of the central nervous system (CNS) affecting approximately two and a half million individuals worldwide and is currently incurable (17, 18). It is characterized by demyelination of axons, resulting in various neurological and psychiatric symptoms (17). The currently used pharmacological treatments for MS include beta interferons; corticosteroids; i.v. immunoglobulins; dimethyl fumarate; glatiramer acetate; the dihydroorotate dehydrogenase inhibitor, teriflunomide; the purine analog, cladribine; the sphingosine-1-phosphate receptor modulators, fingolimod, ozanimod, and siponimod; the anti–α4-integrin antibody, natalizumab; the anti-CD20 antibodies, ocrelizumab, rituximab, and ofatumumab; and the anti-CD52 antibody, alemtuzumab (19, 20, 21). All of these treatments have only limited efficacy and significant safety issues, which imposes the urgent need for the development of novel therapeutic approaches (22). Plexin-B1 is expressed on microglia, a resident macrophage population in the brain, which is increasingly recognized as a pharmacological target in MS (23). In experimental autoimmune encephalomyelitis (EAE), a mouse model of MS, it has been demonstrated that T cells infiltrating the CNS express Sema4D (24). Sema4D-induced activation of Plexin-B1 results in the release of reactive nitric oxide species from microglia, known to promote demyelination and axonal degeneration (25). Consistently, neuroinflammation and disease progression of EAE are strongly reduced in mice lacking Sema4D or Plexin-B1 (24).

Given the pathophysiological importance of the Sema4D–Plexin-B1 interaction in mouse models of osteoporosis and MS, a pharmacological intervention to block their interaction could represent a promising therapeutic approach to treat osteoporosis and MS. In light of the relatively large binding interface between Sema4D and Plexin-B1 (3), antagonistic antibodies appear as an advantageous therapeutic modality. The phage display technology represents a powerful and established platform for the discovery of therapeutic monoclonal antibodies by means of generation and display of vast antibody repertoires (libraries) on the surface of the M13 phage particles, followed by their selection (panning) against the given target and screening (26). Although the technology was initially established using mouse and human antibody libraries, rabbit immune repertoires have become a very attractive source of therapeutic antibodies due to their unique ontogeny and ability to generate distinctive and potent antibody repertoires (27).

Here, we report the generation and characterization of an antagonistic anti–Plexin-B1 antibody isolated from a single-chain variable fragment (scFv) immune rabbit library, which efficiently blocks the interaction of human Plexin-B1 and Sema4D and inhibits Plexin-B1–mediated cellular effects *in vitro*. By employing a mouse line expressing human instead of endogenous murine Plexin-B1, we demonstrate that this anti–Plexin-B1 antibody exhibits therapeutic efficacy in preclinical models of osteoporosis and MS *in vivo*. Finally, we also describe the humanization and developability assessment of this antibody. Taken together, this work establishes a monoclonal anti–Plexin-B1 antibody as a novel potential pharmacological agent for the treatment of osteoporosis and MS.

## Results

### Generation and binding characterization of the anti–Plexin-B1 antibody PLX7

**Figure 1:**
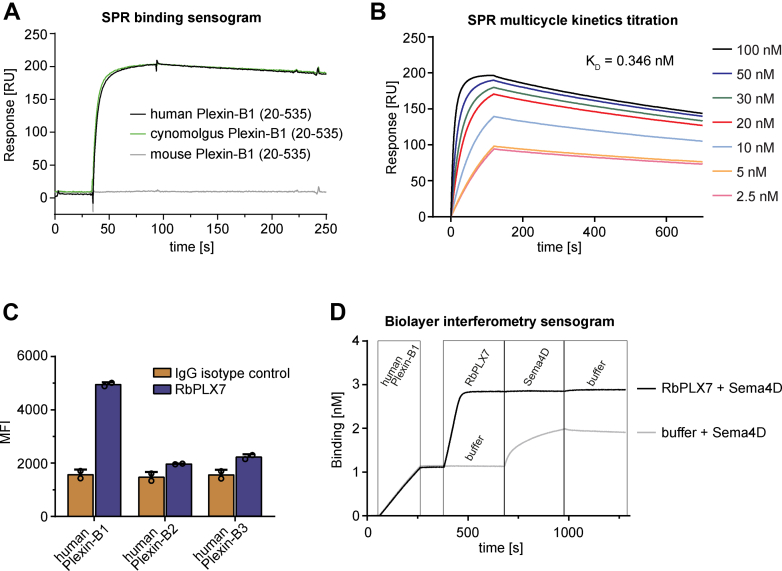
**Binding characteristics of RbPLX7**. *A*, SPR-binding sensogram showing binding kinetics of the RbPLX7 antibody (1 μg/ml) to the extracellular domain (20–535) of human (*black line*), cynomolgus (*green line*), and mouse Plexin-B1 (*gray line*) (10 μg/ml). *B*, kinetic-binding curves obtained by SPR multicycle kinetics titration of recombinant human Plexin-B1 (20–535) on the sensor-immobilized RbPLX7 antibody. Individual sensograms (from *top* to *bottom*) correspond to 100, 50, 30, 20, 10, 5, and 2.5 nM of Plexin-B1 (20–535). *C*, binding of RbPLX7 to full-length human Plexin-B1, -B2, and -B3 expressed in Expi293 cells as analyzed by flow cytometry. Human IgG1 represents the isotype control used in each assay. *D*, biolayer interferometry sensogram showing the binding of Sema4D (50 nM) to human recombinant Plexin-B1 (20–535) (85 nM) in the presence (*black sensogram*) and absence (*gray sensogram*) of RbPLX7 (50 nM). Injection points of proteins or buffer are indicated. Representative examples of at least three (Fig. 1, *A*, *B*, and *D*) or 2 (Fig. 1*C*) independent experiments (biological replicates) are shown. MFI, median fluorescence intensity; Sema4D, semaphorin 4D; SPR, surface plasmon resonance.

To raise antibodies against Plexin-B1, two rabbits were immunized with the recombinant extracellular region (residues 20–535) of human Plexin-B1. As assessed by ELISA, both rabbits developed strong and specific responses against the immunogen (Fig. S1, *A*–*D*). Interestingly, a significant cross-reactivity toward the cynomolgus ortholog but not to mouse Plexin-B1 was observed in both rabbits (Fig. S1*E*). A scFv phage library was generated and enriched for anti–Plexin-B1 antibodies (Table S1). After screening and sequencing analysis, representative scFv clones from dominant clusters were reformatted to a chimeric (rabbit/human) IgG1 isotype, and based on (1) screening for binding to human Plexin-B1 (2), cross-reactivity, and (3) the potential to block the interaction with Sema4D (Table S2), the most promising clone, designated PLX7, was selected for further characterization. Due to the rabbit origin and characteristic presence of noncanonical germline cysteine residues in variable domains of the PLX7 clone, we decided to address these potential liabilities early by substituting two cysteines with serine residues at position 55 and 96 (IMGT numbering) of VH and VL domains, respectively. The Cys-engineered version of chimeric (rabbit/human) PLX7 was designated as RbPLX7 and used for further experiments. First, the purified RbPLX7 clone was evaluated for binding affinity and specificity using several *in vitro* methods. In surface plasmon resonance (SPR) measurements, a single point injection of Plexin-B1 orthologs over immobilized RbPLX7 showed highly similar kinetic sensograms for human and cynomolgus Plexin-B1, in contrast to a complete lack of binding to mouse Plexin-B1 (Fig. 1*A*). This specificity profile correlated well with the reactivity of the polyclonal rabbit sera (Fig. S1*E*). Analysis of binding kinetics and affinity using SPR revealed a subnanomolar affinity of RbPLX7 toward the recombinant extracellular region (residues 20–535) of human Plexin-B1, characterized by fast k_a_ (1.49 × 10^6^ M^−1^s^−1^) and slow k_d_ (5.16 × 10^−4^ s^−1^), with a calculated K_D_ value of 3.46 × 10^−10^ M (Fig. 1*B*). Next, we analyzed binding of RbPLX7 to cells expressing full-length human Plexin-B1 by flow cytometry and examined a potential cross-reactivity with two other plexins of the same sub-family, that is, Plexin-B2 and Plexin-B3. The results confirmed binding of the antibody to full-length human Plexin-B1 and also demonstrated high selectivity with no detectable binding to Plexin-B2 or Plexin-B3 (Fig. 1*C*). Finally, we demonstrated that RbPLX7 interferes with binding of Sema4D, the high-affinity ligand of Plexin-B1, to the recombinant extracellular domain of human Plexin-B1 (Fig. 1*D*). Taken together, these data show that RbPLX7 binds to human Plexin-B1 with high affinity and specificity and blocks the interaction of Plexin-B1 with its ligand, Sema4D.

### RbPLX7 blocks Sema4D-induced plexin-B1–mediated cellular effects in COS-7 cells and primary osteoblasts

**Figure 2:**
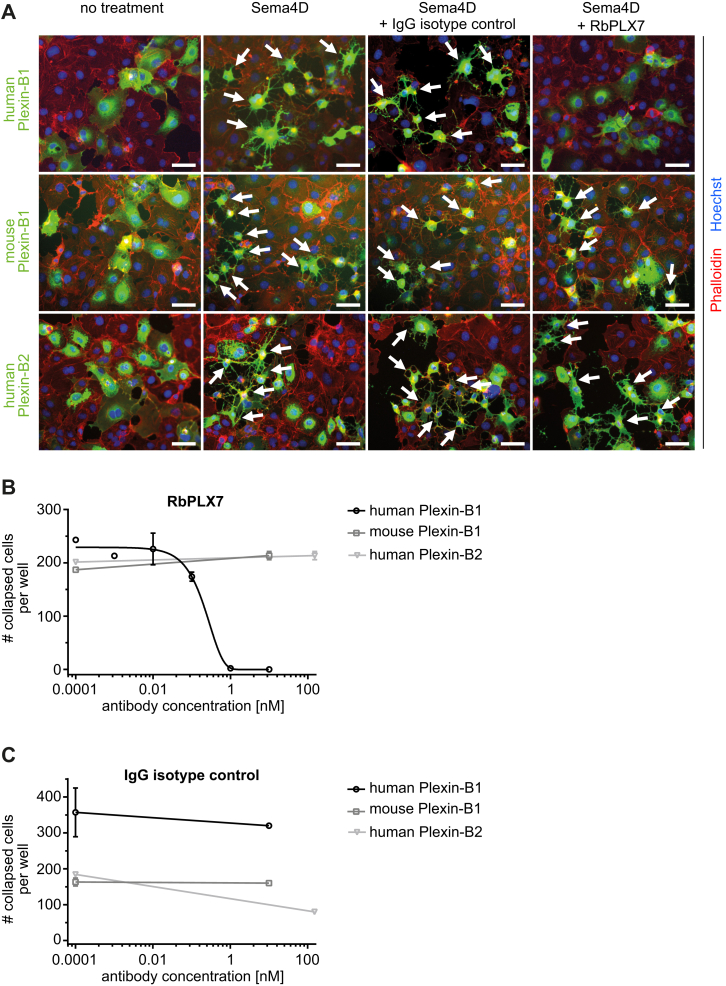
**RbPLX7 blocks Sema4D-induced COS-7 cell collapse.***A*, COS-7 cells were transfected with cDNA encoding human Plexin-B1-FLAG (human Plexin-B1), mouse Plexin-B1-FLAG (mouse Plexin-B1), or human Plexin-B2-FLAG (human Plexin-B2). Forty eight hours after cDNA transfection, cells were treated with RbPLX7 or IgG isotype control (10 nM for Plexin-B1–expressing cells and 150 nM for Plexin-B2–expressing cells), followed by treatment without or with Sema4D (50 nM for Plexin-B1–expressing cells and 150 nM for Plexin-B2–expressing cells). Shown are representative fluorescence images of immunostainings using anti-FLAG antibodies (*green*). *White arrows* indicate examples of collapsed cells. The scale bar represents 50 μm. *B* and *C*, COS-7 cells were transfected as described in (*A*), treated with (*B*) RbPLX7, or (*C*) IgG isotype control at the indicated concentrations followed by Sema4D, and cellular collapse was determined as described in Experimental procedures. Shown are representative examples of two independent experiments (biological replicates). Graphs depict mean values ± s.d. Sema4D, semaphorin 4D.

**Figure 3:**
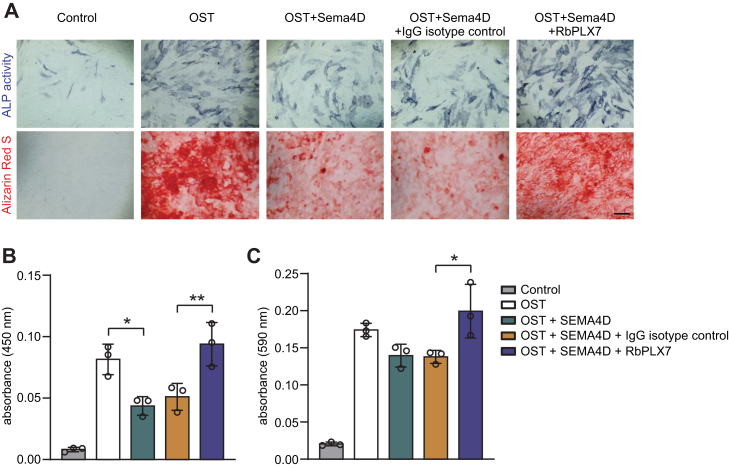
**RbPLX7 blocks Sema4D-induced inhibition of osteoblast differentiation and mineralization.***A*, human osteoblasts were grown in osteoblast growth medium (control) or in osteogenic medium (OST) to induce differentiation and mineralization. Osteoblasts were then exposed to 150 nM of Sema4D in osteogenic medium without or in combination with 150 nM anti–Plexin-B1 antibody RbPLX7 (RbPLX7) or the corresponding IgG isotype control antibody (IgG). Osteoblast differentiation was determined after 7 days *via* measurement of alkaline phosphatase (ALP) activity, and osteoblast mineralization was analyzed after 21 days *via* Alizarin Red S staining. The scale bar represents 100 μm. *B* and *C*, quantification of the results in (*A*). Shown is a representative example of 3 independent experiments (biological replicates). Graphs depict mean values ± s.d. Sema4D, semaphorin 4D.

To test for the functional activity of RbPLX7 in a biological context, we employed the “COS-7 collapse assay”, a cell-based assay widely used to assess semaphorin–plexin signaling: when engineered to express plexins, COS-7 cells exhibit a morphological collapse in response to semaphorins (28) (Fig. S2), and pharmacological agents that block the semaphorin–plexin interaction would be expected to prevent this morphological change. Indeed, RbPLX7 efficiently inhibited Sema4D-induced cellular collapse, when COS-7 cells expressed human Plexin-B1 (Fig. 2 and Table S3). In contrast and in full agreement with the biochemical binding assays described above, RbPLX7 did not interfere with the Sema4D-induced COS-7 collapse mediated by mouse Plexin-B1 or human or mouse Plexin-B2 (Fig. 2 and Table S3). Next, we studied the impact of RbPLX7 on primary human osteoblasts. Consistent with published results (15), Sema4D suppressed osteoblast differentiation and mineralization, as assessed by alkaline phosphatase activity and Alizarin Red S staining, respectively. Fully in line with its ability to inhibit binding of Sema4D to Plexin-B1, RbPLX7 entirely blocked these effects (Fig. 3). Taken together, these data show that Sema4D-induced Plexin-B1–mediated cellular responses are efficiently blunted by RbPLX7.

### Generation of a humanized Plexin-B1 mouse line

Next, we aimed to analyze the effects of the monoclonal anti–Plexin-B1 antibody RbPLX7 in *in vivo* models of osteoporosis and MS. Given the high selectivity of RbPLX7 for human Plexin-B1 with no significant affinity to mouse Plexin-B1 (see above), the use of standard mouse models was precluded. To circumvent this limitation, we generated a genetically-modified mouse line, which expresses human instead of endogenous murine Plexin-B1. To do so, we first generated BAC transgenic mice which express human Plexin-B1. This mouse line was crossed twice with mice heterozygous for the *plxnb1* classical knockout allele (*plxnb1*^+/−^), in which the endogenous murine *plxnb1* locus has been inactivated by gene targeting (29). Using this strategy, we obtained mice, in which the transgenic BAC-encoded human *plxnb1* allele was the only functional copy of the *plxnb1* gene (BAC human *plxnb1*; *plxnb1*^−/−^). To validate this mouse line, which we here term “humanized Plexin-B1 mouse line”, we first analyzed the expression of human and murine Plexin-B1 in bone tissue and primary microglia. While the expression of murine *plxnb1* mRNA was lost (Fig. S3*A*), these mice expressed human *plxnb1* mRNA (Fig. S3, *B* and *C*). Also on the protein level, human Plexin-B1 became detectable in primary microglia (Fig. S3*D*). To assess whether expression of human Plexin-B1 in the humanized Plexin-B1 mouse line was functionally equivalent to the expression of endogenous murine Plexin-B1, EAE experiments were performed. Indeed, and highly comparable to WT mice expressing endogenous murine Plexin-B1 (24), humanized Plexin-B1 mice displayed more pronounced clinical symptoms than mice lacking Plexin-B1 (Fig. S3*E*). In summary, these results establish the humanized Plexin-B1 mouse line as a valid approach to study the function of Plexin-B1 in disease models.

### Therapeutic effects of RbPLX7 in mouse models of postmenopausal osteoporosis and MS

**Figure 4:**
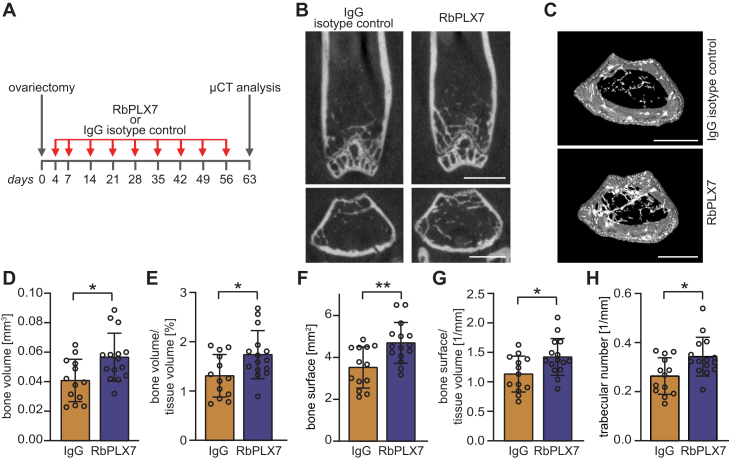
**Therapeutic effects of RbPLX7 in a mouse model of postmenopausal osteoporosis.***A*, humanized Plexin-B1 mice were ovariectomized and treated with the anti–Plexin-B1 antibody RbPLX7 or the corresponding IgG isotype control antibody at the indicated time points. *B*, microcomputed tomography (μCT) and morphometric analysis of the distal femur of humanized Plexin-B1 mice treated with RbPLX7 or with the corresponding IgG isotype control antibody. *Top*: longitudinal view; *bottom*: transaxial view of the metaphyseal region. *C*, cross sectional 3D μCT images of the distal femoral metaphyseal region. *D*–*H*, quantification of the results in (*B*). Shown are mean values ± s.d.; mice treated with IgG isotype control: n = 13; mice treated with RbPLX7: n = 15. The scale bar represents 1 mm.

**Figure 5:**
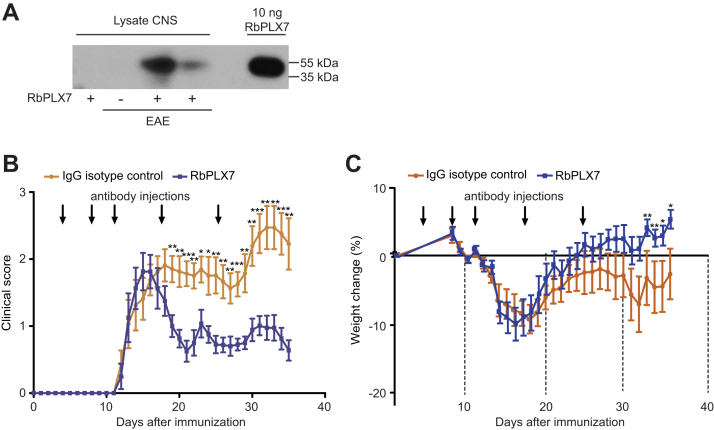
**Therapeutic effects of RbPLX7 in a mouse model of multiple sclerosis.***A*, penetration of RbPLX7 into the central nervous system (CNS). Healthy mice or mice with experimental autoimmune encephalomyelitis (EAE) were injected without or with RbPLX7 according to the schemes depicted in panel (*B*) and (*C*). At day 26 (24 h after the last antibody injection), mice were perfused with PBS, spinal cords were harvested and lysed. Each lane represents the spinal cord lysate of an individual mouse. Western Blot analysis was performed by using an anti-human Fc-antibody. *B* and *C*, clinical score and weight change of humanized Plexin-B1 mice with EAE treated with IgG isotype control (IgG) or the anti–Plexin-B1 antibody RbPLX7 (RbPLX7) over the course of 35 days. Shown are mean values ± s.e.m.; mice treated with IgG: n = 8; mice treated with RbPLX7: n = 8.

We then asked whether an inhibition of the Sema4D–Plexin-B1 interaction by RbPLX7 could be therapeutically harnessed. First, we examined the effect of RbPLX7 in a mouse model of postmenopausal osteoporosis. Female mice were ovariectomized and treated with intravenous injections of RbPLX7 over the course of 8 weeks (Fig. 4*A*). As revealed by microcomputed tomography, RbPLX7 administration exerted a protective effect on bone loss after ovariectomy (Fig. 4, *B*–*H*). No adverse effects associated with the administration of RbPLX7 were observed. Next, we analyzed the therapeutic potential of RbPLX7 in EAE. While not crossing the blood-brain barrier under normal physiological conditions, RbPLX7 efficiently penetrated into the CNS during neuroinflammation (Fig. 5*A*). Consistently, intravenous injections of RbPLX7 significantly improved clinical symptoms (Fig. 5*B*) and alleviated weight loss (Fig. 5*C*) of EAE mice. Taken together, these data demonstrate therapeutic efficacy of RbPLX7 in mouse models of postmenopausal osteoporosis and MS.

### Humanization and developability assessment of PLX7

**Figure 6:**
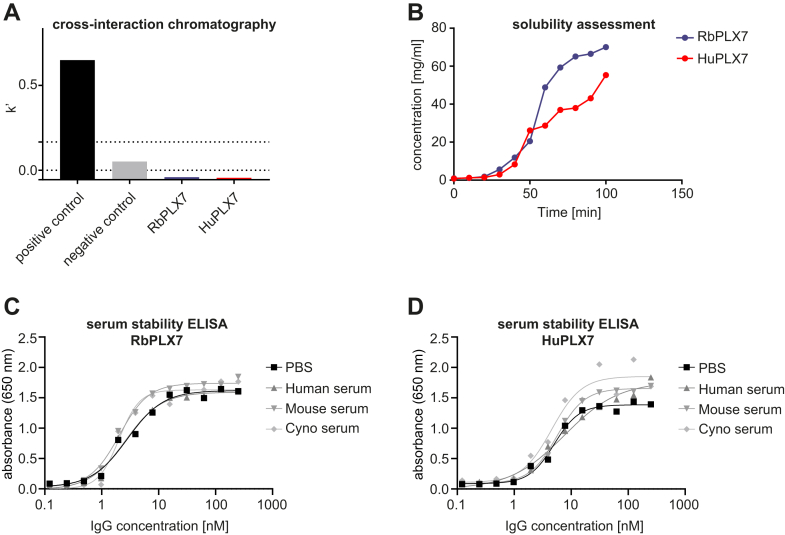
**Biophysical assessment of RbPLX7 and HuPLX7.***A*, cross-interaction chromatography (CIC) assay results showing calculated retention factors (k’) plotted for RbPLX7, HuPLX7, and control antibodies. The two *dotted lines* correspond to k’< 0.05 (desirable) and k’ < 0.2 (acceptable) thresholds, respectively. *B*, solubility assessment by the solvent absorption method. The concentration values (mg/ml) are plotted against the corresponding time points to generate the concentration profiles. *C* and *D*, ELISA assays showing binding profiles of (*C*) RbPLX7 and (*D*) HuPLX7 to human Plexin-B1 (20–535) after 31 days incubation in PBS, mouse, human, and cynomolgus (*cyno*) serum.

To evaluate if the PLX7 antibody could be developed further and meet developability criteria, the chimeric RbPLX7 antibody was humanized by grafting complementarity-determining regions (CDRs) onto donor–human variable domain frameworks, IGHV3-30∗03 for heavy and IGKV1-5∗01 for light kappa chain. A range of humanization variants with rabbit backmutations were tested and the best performing humanization variant was selected and designated as HuPLX7. To confirm that the properties of RbPLX7 were retained by the humanized HuPLX7, we measured its binding characteristics and functional activity. The results summarized in Table S4 demonstrate that despite a reduced affinity (∼4.5-fold), HuPLX7 efficiently blocked Sema4D–Plexin-B1 binding. Moreover, HuPLX7 showed a ∼2.5-fold increase in expression yield (Table S4), which represents one of the key parameters for defining a “drug-like” profile of any therapeutic candidate. Next, we performed a range of biophysical tests to further evaluate developability attributes like aggregation, conformational stability, solubility, nonspecific interactions, and serum stability. To do so, we used a SEC-MALS analysis to assess the aggregation propensity under normal and stress conditions like freeze-thaw and elevated temperature. The results show that both, RbPLX7 (rabbit/human chimeric) and HuPLX7 (humanized), were monodispersed with no significant aggregation under normal and stress conditions (Table S5). In addition, a cross-interaction chromatography analysis was carried out to assess proneness to nonspecific protein–protein interactions and to probe for solubility issues, which could interfere with downstream manufacturing processes. As shown in Figure 6*A*, both antibodies displayed low retention indices, indicating low self-interaction propensities and good solubilities. The solubility was further examined by solubility assessment showing that both versions of PLX7 are not prone to precipitation at concentrations up to 70 mg/ml (Fig. 6*B*). Finally, we assessed physicochemical stability in the presence of serum showing that both versions of PLX7 retained their binding to human Plexin-B1 after exposure to human, mouse, and cynomolgus sera at 37 °C for 31 days (Fig. 6, *C* and *D*). In summary, these data demonstrate that the humanized monoclonal anti–Plexin-B1 antibody HuPLX7 fulfills key biophysical characteristics of a therapeutic drug.

## Discussion

Osteoporosis and MS are both highly prevalent diseases with clearly insufficient treatment options. Here, we describe the generation, characterization, and humanization of a novel monoclonal antibody directed against the transmembrane receptor Plexin-B1 that exerts therapeutic effects in mouse models of osteoporosis and MS with a mechanism distinct from all approved drugs.

In MS, microglia, and in particular the interaction of microglia with T cells, play a pivotal role in pathogenesis (30, 31, 32, 33). The activation of Plexin-B1 on microglia by Sema4D on T cells has been shown to trigger microglia activation (24). Our results now demonstrate that a block of Sema4D binding to Plexin-B1 by the anti–Plexin-B1 antibody RbPLX7 exerts therapeutic effects in EAE. Several drugs currently used in the treatment of MS—including dimethyl fumarate, the sphingosine-1-phosphate receptor modulator fingolimod, the anti–α4-integrin antibody natalizumab, and the anti-CD20 antibody ofatumumab—are associated with an increased risk for progressive multifocal leukoencephalopathy (34). This devastating CNS disease is caused by the JC virus, which establishes persistent asymptomatic infections in immune-competent hosts but can be reactivated when functions of T lymphocytes are impaired (35). Given that Plexin-B1 is expressed on microglia (24), rather than on lymphocytes (36, 37), it seems reasonable to assume that a therapy based on Plexin-B1 blockade is not expected to carry the risk of progressive multifocal leukoencephalopathy.

For decades, the treatment of osteoporosis has relied on antiresorptive drugs, in particular bisphosphonates (13, 14). However, instead of promoting bone formation, these drugs support a relative increase in bone mass by suppressing bone resorption of osteoclasts and are associated with rare but significant adverse effects (13, 14). Anabolic drugs—either alone or in combination with antiresorptive drugs—represent a novel therapeutic approach for osteoporosis by enhancing bone formation (13, 14). As genetic deletion of Sema4D or Plexin-B1 results in elevated bone mass in mice by promoting osteoblastic bone formation without affecting osteoclastic bone resorption (15), pharmacological inhibition of Plexin-B1 could potentially add a new anabolic principle to osteoporosis treatment. Indeed, our study reveals that the anti–Plexin-B1 antibody RbPLX7 exerts beneficial effects in a mouse model of postmenopausal osteoporosis. These findings are fully in line with a recently published report, demonstrating *in vivo* osteogenic activity of a cyclic peptide that inhibits Plexin-B1 signaling (38). Interestingly, this cyclic peptide exerts an allosteric mechanism of Plexin-B1 inhibition (39). In contrast, the PLX7 antibody is likely to sterically block the interaction between Plexin-B1 and its ligand, Sema4D; however, more data about the binding epitope of PLX7 will be required to determine its exact mechanism of action.

Of note, MS is known to be associated with an increased risk of osteoporosis and bone fractures (40). The etiology of osteoporosis in MS patients is most likely multifactorial with immobility, vitamin D deficiency, and therapeutic use of glucocorticoids representing possible contributing factors (41), all of which negatively affect osteoblast function (42, 43, 44). Therefore, a pharmacological Plexin-B1 blockade in MS patients could potentially ameliorate both neuroinflammation as well as MS-associated osteoporosis.

It has been previously shown that the administration of an anti-Sema4D antibody inhibits neuroinflammation during EAE development (24, 45) and is protective against bone loss in a mouse model of postmenopausal osteoporosis (15). These effects are similar to those induced by an anti–Plexin-B1 antibody as reported here, underlining the functional importance of Sema4D-Plexin-B1–mediated osteoclast–osteoblast and T cell–microglia communication. However, it is noteworthy that in addition to Plexin-B1, Sema4D is known to activate other receptors, that is, Plexin-B2, which is widely expressed on epithelial cells (46, 47) and CD72, which serves as receptor on lymphocytes (36, 37). Conversely, Plexin-B1 has been demonstrated to bind to other semaphorins in addition to Sema4D, that is, to Sema3C and Sema4A (48, 49), which is of particular relevance in cancer cells: the Sema3C-induced activation of Plexin-B1 on prostate cancer cells promotes cancer growth through transactivation of receptor tyrosine kinases, such as the EGF receptor, ErbB-2 (HER2), and the hepatocyte growth factor receptor (c-Met) (48); the binding of Plexin-B1 to transmembrane Sema4A on cancer and on dendritic cells triggers a reverse signaling pathway, which controls cell migration *via* the scaffold protein Scrib (50). Of note, Sema4D and Sema4A have been shown to compete for binding to Plexin-B1 (51), suggesting that they share similar binding interfaces on Plexin-B1; this implicates that RbPLX7 might block binding of Plexin-B1 to both Sema4D as well as to Sema4A. In summary, it seems more than likely that the biological effects of anti-Sema4D and anti–Plexin-B1 antibodies are overlapping but are not identical.

In addition to demonstrating beneficial effects of the rabbit/human chimeric RbPLX7 antibody in *in vivo* disease models of osteoporosis and MS, we report its successful humanization and preliminary biophysical characterization, which are critical to evaluate its developability potential. Our results show an attractive biophysical profile of the humanized HuPLX7 antibody fulfilling key criteria for expression yield, stability, aggregation, and nonspecific interactions. These promising findings call for the continuation of the preclinical stage assessment including PK characterization, early toxicity testing, and off-target screening to further establish HuPLX7 as a therapeutic candidate. Given that the mechanism of action of HuPLX7 is expected to be through steric hindrance of the Plexin-B1–Sema4D interaction and not to be dependent on Fc-mediated effector functions, the conversion of HuPLX7 from the IgG1 to the IgG4 isotype would be desirable before any preclinical toxicity studies to minimize potential toxicity effects mediated by antibody-dependent cell-mediated cytotoxicity and complement-dependent cytotoxicity. Moreover, the inclusion of an additional control could be advantageous during the preclinical phase. Such a control could be a HuPLX7 mutant, which harbors specific mutations preventing its binding to Plexin-B1; however, the design of such mutations would require a detailed characterization and understanding of the binding interface between HuPLX7 and Plexin-B1. For some of the studies mentioned above, our newly generated humanized Plexin-B1 mouse line could serve as a valuable *in vivo* model.

In summary, we here describe the generation and characterization of an anti–Plexin-B1 antibody that specifically interferes with binding of Plexin-B1 to its high-affinity ligand, Sema4D, and validate its therapeutic efficacy in preclinical mouse models of osteoporosis and MS. Given that cell–cell communication *via* Sema4D–Plexin-B1 signaling has been shown to be also functionally relevant in several other pathophysiological conditions such as diabetic retinopathy (52) and cancer (53, 54, 55, 56), it is tempting to speculate that the anti–Plexin-B1 antibody PLX7 could hold therapeutic potential in additional disease contexts.

## Experimental procedures

### Generation of the extracellular domain of Plexin-B1

A human Plexin-B1 (1–535) fragment was cloned into the pcDNA5 vector using HindIII (R0104S, NEB) and XhoI (R0146S, NEB) restriction sites with the addition of a C-terminal 6xHis tag and expressed in Expi293F cells (Invitrogen) according to the manufacturer’s instructions. Supernatant-containing secreted Plexin-B1 (20–535)-6xHis was collected after 7 days and captured on an Excel HisTrap (GE) column in 20 mM Hepes, pH 8.0, 0.3 M NaCl, and 10 mM imidazole buffer. After washing the column with 10 column volumes of washing buffer (20 mM Hepes, pH 8.0, 0.3 M NaCl, and 20 mM imidazole), the protein was eluted with elution buffer (20 mM Hepes, pH 8.0, 0.3 M NaCl, and 250 mM imidazole). The eluted fraction was further purified by gel filtration using a Superdex 200 16/60 column equilibrated with PBS. Collected fractions were analyzed by SDS-PAGE, pooled, quantified, aliquoted, and flash-frozen for storage at −80 °C. The same method was used to generate corresponding fragments of mouse and cynomolgus Plexin-B1—mouse Plexin-B1 (20–535)-6xHis and cynomolgus Plexin-B1 (20–535)-6xHis, respectively. An Avi-tagged version of human Plexin-B1 (20–535) was generated by insertion of the Avi-tag (GLNDIFEAQKIEWHE) between the C-terminus of the Plexin-B1 fragment and the 6xHis-tag using the QuikChange Lightning Site-Directed Mutagenesis Kit (# 210518, Agilent Technologies). Human Plexin-B1 (20–535)-Avi-6xHis was expressed and purified using the same method as described for the non-Avi tag version. The *in vitro* biotinylation of the Avi-tagged Plexin-B1 was carried out using the BirA enzyme (BirA500 standard reaction kit, Avidity) according to the manufacturer’s protocol. Free biotin was removed by a desalting method using Zeba Spin 7K desalting columns (89889, ThermoFisher). The biotinylation of Plexin-B1 was verified by Western Blotting analysis using Streptavidin-HRP conjugate (N100, ThermoFisher).

### Rabbit immunization

Protein immunization was performed by BioGenes Antibodies (Germany). The purified antigen, that is, human Plexin-B1 (20–535)-6xHis was injected four times to each rabbit at day 0, 7, 14, and 21, using a fast 35 days immunization schedule. Preimmune, intermediate, and final serum was collected at day 0, 21, and 35, respectively. The spleen was extracted and stored in RNAlater reagent (AM7020, ThermoFisher). Sera of individual rabbits were analyzed for antigen-specific responses by a standard ELISA assay using the biotinylated human Plexin-B1 (20–535)-Avi-6xHis. Briefly, HBC Streptavidin plates (Pierce) were coated with 50 μl of 5 μg/ml of biotinylated antigen diluted in PBS. After washing, 50 μl of sera diluted in PBS were added and incubated for 1 h, followed by incubation with goat anti-rabbit-HRP conjugate (65–6120, Invitrogen) for 1 h. Fifty microliters of TMB substrate was added and the reaction was stopped with 50 μl of 0.5 M H_2_SO_4_. The absorbance was recorded at 450 nm and the data were fitted to 4-parameter dose response equation using GraphPad Prism 7.0.1 software (https://www.graphpad.com/scientific-software/prism/). For cross-reactivity assessment against cynomolgus and mouse orthologs, a similar ELISA assay was carried out. In this case, diluted sera were preincubated with 20 μg/ml of mouse or cynomolgus Plexin-B1 (20–535)-6xHis before adding to the plate coated with the biotinylated human Plexin-B1 (20–535)-Avi-6xHis.

### Generation of a rabbit immune phage display library

Based on a slightly higher specific response combined with lower nonspecific background, rabbit 88 (Rb88) was chosen to generate the immune scFv phage library. To do so, total RNA was extracted from rabbit’s spleen using the RNeasy Maxi kit (75162, Qiagen) according to the manufacturer’s protocol. DNA was synthesized using the SuperScript III First-Strand Synthesis System (18080–051, Invitrogen) with oligo (dT)_20_ primers according to manufacturer’s protocol. The gene fragments encoding antibody variable domains (VH and VL) were amplified from cDNA using germline-specific oligos listed in the Table S6. Four primer pairings for VH and four for VL were used for the individual first-PCR reactions, each containing PhusionFlash polymerase master mix (ThermoFisher, F-548L) and subjected to 30 cycles at 98 °C for 1 s, 55 °C for 5 s, 72 °C for 15 s, followed by the final incubation at 72 °C for 1 min. Bands corresponding to VH and VL domains (∼350–400 bp) were purified from a 2% agarose gel employing the Qiagen gel extraction kit (28704, Qiagen) and used for the second-PCR reaction with primers incorporating cloning sites and the synthetic linker (Table S7). The gel-purified VH and VL repertoires were assembled into scFv library format by two-stage overlap extension PCR using PhusionFlash polymerase mastermix, run for 10 cycles at 98 °C for 1 s, 60 °C for 5 s, 72 °C for 15 s followed by a further 25 cycles after addition of Rb-PTH-For and Rb-PTL-Not-Rev primers (Table S8). The gel-purified scFv fragment was digested with SfiI (NEB, R0123S) and NotI (NEB, R3189M) and ligated into the SfiI/NotI–digested pHEN1H6 phagemid vector. The ligated products were electroporated into electrocompetent TG1 cells (Lucigen, 60502) and plated onto 2xYTAG agar (2xYT media supplemented with 100 μg/ml ampicillin, 2% glucose) bioassay plates. Bacterial colonies were scraped from plates and the generated library stored at −80 °C in the presence of 20% glycerol. The library had an estimated size of 4 × 10^7^ individual clones. To assess its quality, 96 random clones were sequenced and analyzed, showing low levels (∼3%) of nonproductive clones and high diversity—99% unique clusters for both CDR3-H and CDR3-L.

### Phage library selection

Before antigen biopanning, the phage library was rescued by M13KO7 helper phage (NEB, #N0315) using 1:20 cells/phage ratio, and phage particles precipitated with 20% PEG 8000/2.5 M NaCl solution were purified using a standard protocol (57). Three rounds of “in solution” panning were performed using streptavidin (SA)-coated Dynabeads M-280 (11205D, ThermoFisher). Briefly, 10^11^-10^12^–purified phages were incubated (blocked) in 2% milk/PBS for 1 h at room temperature to block nonspecific interactions. The same incubation was performed for the Dynabeads M-280. For round one of biopanning, the blocked phage library was mixed with biotinylated human Plexin-B1 (20–535)-Avi-6xHis (final antigen concentration of 10 nM). After 1 h incubation at room temperature, the magnetic beads were added to capture the biotinylated target. Beads were pelleted after 15 min incubation and washed six times with PBS/0.1% Tween solution before bound phages were eluted with 100 mM triethylamine (T0886, Sigma-Aldrich) and used for infection of TG1 cells (in logarithmic growth phase). The infected TG1 cells were plated on 2xYTAG agar bioassay plates and collected the next day, providing an enriched library for the next rounds of the biopanning. The same steps were performed for subsequent rounds, however, with lower target concentrations, that is, 5 nM and 0.5 nM in round two and round three, respectively. A 277-fold enrichment was observed after the second round of selection and no further increase was observed in the third round. Importantly, no significant enrichment was observed in the parallel nontarget biopanning using SA beads only.

### Phage ELISA

Random clones from the enriched library after round two and three were picked, and phage-containing supernatants were produced using a standard method ((Coomber D, Methods in Molecular Biology Eds O’Brien PM and Aitken R) vol. 178 (2002)) and subjected to antigen-binding standard ELISA assay. Briefly, 96-well Nunc-Immuno Maxisorp plates (442404, ThermoFisher) were coated with 3 μg/ml SA (21122, ThermoFisher) and incubated overnight at 4 °C. Fifty microliters of 1 μg/ml biotinylated antigen (or nonrelevant target) was added. After 30 min incubation, plates were washed with PBS/0.1% Tween and blocked with 2% milk/PBS for 1 h at room temperature. Fifty microliters of prepared phage supernatants (blocked for 1 h with 2% milk/PBS) was added to each well and incubated for 1 h at room temperature. After washing with PBS/0.1% Tween, the M13-HRP antibody (GE, 27-9421-01) diluted in 2% milk/PBS (1/5000) was added to each well and incubated for 30 min. Finally, bound phages were detected with TMB substrate (Sigma, T4444) and colorimetric reactions quenched by 0.5 M sulfuric acid. Absorbance was measured at 450 nm. Screening of random clones from each biopanning round showed the large number of Plexin-B1–specific clones with the steep increase from 6% after first round to 87% after second round. Single clones on 96-well plates were subjected to Sanger sequencing performed by GATC. Sequencing analysis of CDR3-H diversity revealed the presence of several distinct clusters with some clones present in high proportion especially after the third round. To avoid any further diversity loss, no additional panning round was considered.

### Generation of chimeric (rabbit/human) IgGs

Based on the sequencing analysis, representative clones were selected and reformatted to rabbit/human chimeric IgG1 isotype format comprised of rabbit variable (VH and VL) and human constant domains of IgG1/kappa isotype. Construction of chimeric expression vectors was carried out by cloning of the rabbit heavy and light chain variable regions into separate vectors containing human constant domains, pHuG1 (IgG1 isotype) and pHuK (kappa), respectively, using ligase-independent cloning and transformed into chemically competent TOP10 cells (C404010, ThermoFisher). Several clones were isolated, prepped using the QIAprep Spin Miniprep Kit (27103, QIAGEN), and sequences were verified by Sanger sequencing. To produce chimeric IgG candidates, a pair of plasmids encoding heavy and light chains for each clone was cotransfected into Expi293 suspension cells using ExpiFectamine293 reagent (The Expi293 Expression System Kit, Invitrogen—manufactures standard instructions). After 5 days of cultivation, culture supernatants were collected, and IgG concentration was quantified by OctetRED384 (ForteBio). Supernatants from large-scale cultures were purified by standard antibody purification methods using Protein A or G affinity chromatography followed by size-exclusion chromatography. Concentrations of the purified antibodies were measured by absorbance at 280 nm.

### Binding kinetics by SPR

Binding kinetics and affinity analysis of PLX7 antibodies were performed by SPR using a Biacore T200 and 2000 instruments. For single concentration cross-reactivity testing, 1 μg/ml of antibody was captured on an SA chip coated with Protein A in 1xHBS-P+ buffer, and 10 μg/ml of each plexin ortholog was subsequently injected following a chip regeneration step after each injection using 10 mM glycine, pH 2.0 buffer. For the K_D_ measurement assay, 0.5 μg/ml of antibody was captured on a Protein A chip in 1xHBS-P+ buffer, and a series of Plexin-B1 (20–535)-6xHis concentrations (100, 50, 30, 20, 10, 5, and 2.5 nM) were injected over the flow cell to carry out kinetics measurements. After each dissociation stage, the chip was regenerated with 10 mM glycine, pH 2.0. Experimental data were fitted by 1:1 Langmuir model using BIAevaluation 3.0 software to calculate K_D_ values.

### Cross-reactivity and Sema4D competition assays

Cross-reactivity and capability of anti–Plexin-B1 antibodies to block the interaction between Plexin-B1 and Sema4D was analyzed by biolayer interferometry using OctetRED384 (ForteBio). Briefly, an SA biosensor (ForteBio) was used to capture 5 μg/ml of biotinylated human or mouse Plexin-B1 (20–535)-Avi-6xHis in 1xKinetics buffer (ForteBio). In the next step, the binding response was measured to 50 nM of the antibody or buffer, followed by 50 nM of human Sema4D (7470-S4, R&D). Qualitative analysis of individual binding association curves was performed using the Data Analysis 10.0 software (ForteBio).

### Binding to full-length Plexin-B1 expressed on Expi293F

Expi293F cells were plated into 24-well plates (2.5 × 10^6^ cells per well in a total volume of 845 μl per well) and then transiently transfected with human Plexin-B1-FLAG, mouse Plexin-B1-FLAG, human Plexin-B2-FLAG, or human Plexin-B3-FLAG pcDNA3.1 plasmids (GenScript), using the ExpiFectamine (Gibco, A14524) transfection reagent according to the manufacturer’s instructions. Briefly, 1 μg DNA was mixed with 2.7 μl ExpiFectamine in serum-free medium (OptiMem, Gibco, 31985) in a total volume of 100 μl per well. Following a 20 min incubation, the DNA mixture was added to cells, which were then cultured in a shaker incubator at 225 rpm overnight. The following day, 5 μl of Enhancer 1 and 50 μl of Enhancer 2 were added. Forty eight hours after transfection, cells were harvested, resuspended in PBS buffer (Gibco, 10010015), substituted with 0.1% bovine serum albumin (BSA, Sigma) and transferred into 96-well U-bottom plates (Corning, 3799) (2.5 × 10^5^ cells per well in a total volume 50 μl per well). Then anti–Plexin-B1 antibodies or the isotype control at a final concentration of 10 μg/ml were added to cells. After 30 to 60 min incubation on ice, cells were washed in PBS/0.1% BSA buffer and incubated with secondary AlexaFluor 488 anti-human antibody (Life Technology, A11013) for 30 min at room temperature. Three micromolar of fluorescent dye Draq7 (Biostatus, DR7 1000) were added to cells in the last 10 min of the secondary antibody incubation in order to label dead cells. Following washing with PBS/0.1% BSA, cells were analyzed on an IntelliCyt iQue Screener instrument (Sartorius). Median fluorescence intensity was calculated with ForeCyt 6.2 (R3) software (https://www.sartorius.com/en/products/flow-cytometry/flow-cytometry-software).

### COS-7 collapse assay

Human and mouse Plexin-B1-FLAG and human and mouse Plexin-B2-FLAG pcDNA3.1 plasmids were ordered from GenScript. COS-7 cells were purchased from ATCC and cultured in high-glucose Dulbecco’s modified Eagle’s medium (DMEM) supplemented with GlutaMAX, pyruvate (Gibco, 31966), and 10% fetal bovine serum (Gibco, 16250078). Cells were cultured at 37 °C in 10% CO_2_ and passaged at 80 to 90%. COS-7 cells were seeded in 96-well plates (Greiner, 655090) (6500 cells per well in a total volume of 100 μl per well) and then transiently transfected 24 h later with human or mouse Plexin-B1-FLAG or human or mouse Plexin-B2-FLAG pcDNA3.1 plasmids, using the X-treme Gene 9 transfection reagent according to the manufacturer’s instructions (Sigma, 06365779001). Briefly, 0.05 μg DNA was mixed with 0.2 μl X-treme Gene 9 in serum-free medium (OptiMem, Gibco, 31985) to reach a total volume of 10 μl per well. After 30 min incubation, the DNA mixture was added to cells. After transfection for 48 h, RbPLX7 or the isotype control IgG1k at a concentration of 0.01 to 10 nM (for Plexin-B1) or 150 nM (for Plexin-B2) were added (10 μl/well), incubated for 1 h, followed by an additional 1 h treatment with 50 nM Sema4D (for Plexin-B1) or 150 nM Sema4D (for Plexin-B2). Cells mock-treated with 10 μl medium instead of antibodies were incubated with 0.08 to 50 nM (Plexin-B1) or 10 to 150 nM (Plexin-B2) Sema4D for a Sema4D dose response (10 μl/well) (Fig. S2). The cells were fixed with 4% formaldehyde, permeabilized, and incubated with anti-FLAG antibody (Sigma, F1804) followed by staining with anti-mouse–Alexa Fluor 488 antibody (Life Technology, A11001), Hoechst (Invitrogen, H3570), and phalloidin Texas Red (Life Technology, T7471). After staining, the plates were imaged using a high-content screening system (IN Cell Analyzer 2000, GE Healthcare). Images were analyzed and the number of collapsed cells per well was quantified manually. EC_50_ values were calculated using five-parameters nonlinear regression analysis in GraphPad Prism 8.1.2 (332) software.

### Humanization of RbPLX7

Humanization of RbPLX7 antibody was carried out by the CDR grafting method. Homology models (10) of the variable regions of RbPLX7 were produced using the Bioluminate 1.9 software (Schrodinger) to determine residues which were within 4 Å of the CDR loops. Optimal human frameworks were selected by interrogation of human VH and VL in-house–curated databases using LifeArc’s proprietary antibody sequence analysis software. The selected human VH and VL frameworks were used to design the humanized variants; the first variant is comprised of the IMGT CDRs from RbPLX7 grafted straight into the selected VH and VL frameworks, the second variant comprises the first variant with additional rabbit backmutations at key proximity, VCI, or interface positions, and the remaining variants are composed of the first variant with single rabbit backmutations in key positions. The first and second humanization variants were synthesized (Genscript) and SDM performed on the first variant to produce the remaining variants. The variants were cloned into in-house vectors containing the respective constant regions and expressed using the same method as for chimeric IgGs (described above).

### Freeze-thaw and heat-induced stress tests

Samples were prepared in 1×PBS at 1.0 mg/ml for each stress condition to test along with a 4 °C control sample. Freeze-thaw stress tests were performed by carrying out 10 cycles of 15 min at −80 °C, followed by thawing for 15 min at room temperature. For the heat-induced stress test, the samples were incubated at 4 °C (control), room temperature, 37 °C, and 50 °C for 1 month. The samples were analyzed using SEC-MALS in comparison to the control sample. Briefly, 10 μl of each sample (1 mg/ml) was injected onto an SEC column (Acquity UPLC BEH200 SEC, 4.6 × 150 mm, 1.7 μm) and subsequently detected by three in-series detectors, UV (Agilent 1260 Infinity HPLC system with thermostatted column compartment), light-scattering (Wyatt Technology DAWN HELEOS), and differential refractometer (Wyatt Technology Optilab TRex). A constant flow rate of 0.4 ml/min was applied using a mobile phase of Dulbecco’s PBS (Sigma D8537) containing 0.01% sodium azide. All experiments were carried out at 25 °C. The data was analyzed with Wyatt Technology ASTRA software (version 6.1.2.83) and with the refractive index increment (dn/dc) set to 0.185.

### Cross-interaction chromatography

Samples (0.5 mg/ml) were analyzed by two separate 20 μl injections; firstly, onto a 1 ml NHS-activated resin (GE Healthcare) coupled with 30 mg human polyclonal IgG (Sigma, 14506) and secondly, onto a 1 ml NHS-activated resin blank coupled, as control column. The mobile phase consisted of Dulbecco’s PBS (Sigma D8537) containing 0.01% sodium azide (0.1 ml/min), and all experiments were performed at 25 °C. Eluted samples were detected by UV absorbance (Agilent 1260 Infinity HPLC system with thermostatted column compartment), and data was analyzed using Wyatt Technology ASTRA software (version 6.1.2.83) to determine sample peak retention times. These were then used to calculate a retention factor k’, k’< 0.05 is desirable; k’ < 0.2 is acceptable.

### Solubility assessment

A Vivapore solvent absorption concentrator 7500 kDa MWCO (VP0502 Sartorius) was loaded with 3.5 ml of antibody at 1 mg/ml. Antibody concentration was monitored every 10 min by sampling 3 μl for measuring concentration on the Nanodrop 2000 (ε = 1.4) and continued until the concentrated volume reached a dead volume of ∼30 to 50 μl. The concentration values (mg/ml) were plotted against the corresponding time points to generate the concentration profiles.

### Serum stability assessment

The purified antibody was prepared at 0.4 mg/ml in PBS. Mouse (SCD-808), human (S-123), and cynomolgus (S-118) sera from Seralab were used. Serum and PBS control (150 μl) were aliquoted into a round-bottom 96-well plate (Sigma-Aldrich, CLS3799-50EA), and 50 μl (0.4 mg/ml) antibody solution in PBS was added to the well in triplicates to each serum type in a tissue culture cabinet (BSL-2). The plate was sealed and incubated at 37 °C for 30 days. For the ELISA analysis, samples serially diluted in PBS were incubated on SpectraPLate HB 384-well plates (PerkinElmer, 6007509), coated with 3 μg/ml Streptavidin (ThermoFisher, 21122) followed by 1 μg/ml of biotinylated human Plexin-B1 for 1 h at room temperature. After washing, bound IgG was detected using goat anti-human IgG (Fc specific)−peroxidase conjugate antibody 1:6000 (Sigma, A0170). Plates were washed between each incubation step with PBS-0.5% Tween at room temperature. Finally, K-Blue Substrate (Neogen, 308176) was added to all wells followed by Red Stop solution to stop the colorimetric reaction. Absorbance was read at 650 nm using the Pherastar plus reader (BMG Labtech).

### RT-PCR

To isolate RNA from mouse femora, bones were pestled in liquid nitrogen, homogenized using a planetary ball mill, vortexed in ribozol (VWR) for 45 min at room temperature, and centrifuged. The supernatant was extracted with chloroform. After adding an equal volume of ethanol, RNA was purified using Zymo-Spin IIICG columns (Zymo Research). RNA purification from primary microglia was performed using a Direct-zol RNA Microprep Kit (Zymo Research). BT-474 cells were obtained from the German Collection of Microorganisms and Cell Cultures (DSMZ). cDNA was synthesized by reverse transcription. Quantitative PCR was performed using the Light-Cycler 480 Probes Master system (Roche). The following primers were used: for human *plxnb1*: forward 5′-GACCGAGGTGGCCTACATCGAG-3′ and reverse 5′-ACCTTCAGAAGTGTGCTCTGGGTCATG-3′ (“primer pair #1”), and forward 5′-CGGGACCGCTGCAAGAAGGAATTC-3′ and reverse 5′-TCCACAGTGGGCCGTCTGCTC-3′ (“primer pair #2”); for mouse *plxnb1*: forward 5′-GTGTGCTGGAGCTAGGGAGTCGG-3′ and reverse 5′-CATGCAGCCCATCGGCACTG-3′ (“primer pair #1”), and forward 5′-GCCCGAGGAGCAGCGAGTG-3′ and reverse 5′-TCCTCCCCGCTGGCTCC-3′ (“primer pair #2”).

### Western blotting

Spinal cord tissue was sonicated in ice-cold radioimmunoprecipitation buffer (150 mM NaCl, 50 mM Tris pH 7.4, 5 mM EDTA, 1% Triton X-100, 0.1% SDS, 0.5% sodium deoxycholate, protease inhibitors), tissue lysates were centrifuged, and supernatants were used for Western blotting according to standard laboratory protocols. Anti–human-HRP (Jackson Immuno Research; cat. no. 109-035-098) was used at a dilution of 1:1000.

### Mice

All mice used in this study were on a C57BL/6 genetic background. Mice were housed in individually ventilated cages under a 12-h light-dark cycle with free access to food and water and under specific pathogen-free conditions. Mice were randomized for treatment. Scoring and analysis of mice was done in a blinded manner.

### Generation of “humanized Plexin-B1” mice

The generation of Plexin-B1 KO mice (*plxnb1*^−/−^) was described previously (58). The BAC clone RP11-47K17, carrying the human *plxnb1* gene, was obtained from the BACPAC Resource Center (Children’s Hospital Oakland Research Institute). BAC DNA was linearized by restriction digest, purified using a Sepharose column (Sepharose CL-4B, Sigma), and injected into pronuclei of zygotes (C57BL/6 background). The resulting BAC transgenic mouse line, B6.Cg-Tg(human plxnb1)334383Soff, was crossed twice with the Plexin-B1 KO mouse line (as described above) to obtain mice, which lack a functional endogenous murine *plxnb1* gene, and instead express a transgenic human *plxnb1* gene (an analogous approach for Plexin-B2 and Plexin-D1 has been described (59)). These mice were termed “humanized Plexin-B1” mice. Mice were kept on a C57BL/6 background and heterozygous for the human *plxnb1* transgene. For genotyping, the following primers were used: forward 5′-CTGATACCGGTCCATGTGGAACGC-3′ and reverse 5′-GGAAGCTGGGTCCTGAAGGCTG-3′ (size of PCR products: 231 bp for the endogenous *plxnb1* WT allele, no product for the *plxnb1* KO allele, no product for the transgenic human *plxnb1* allele), forward 5′-GTGGCTTTTCCAGGAGTGTTTGCC-3′ and reverse 5′- GTGGCTCTTCAACAGTCCTTCCG-3′ (size of PCR products: 1649 bp for the endogenous *plxnb1* WT allele, 430 bp for the *plxnb1* KO allele, no product for the transgenic human *plxnb1* allele), forward 5′-GTCGTGTGGTTTGGGGCTGGG-3′ and reverse 5′- GTGTCCTACTTGCTGGCTACTGCAG-3′ (size of PCR products: no product for the endogenous *plxnb1* WT allele, no product for the *plxnb1* KO allele, 291 bp for the transgenic human *plxnb1* allele).

### Osteoblast assays

Human osteoblasts were purchased from PromoCell (cat. no. C-12720) and cultured in osteoblast growth medium (PromoCell, cat. no. C-27001). To induce differentiation, cells were seeded into a 96-well plate (1 × 10^4^ cells/well), and after cells attained confluency, the osteoblast growth medium was replaced by osteogenic medium (PromoCell, cat. no. C-27020). Sema4D (150 nM; R&D Systems, cat. no. 7470-S4-050), anti–Plexin-B1 or IgG control antibodies (150 nM) were added to the osteogenic medium; medium was changed every third day. Alkaline phosphatase activity was analyzed after 7 days, using BCIP/NBT as a substrate (SigmaFast BCIP/NBT, Sigma Aldrich, cat. no. B5655-25TAB). To do so, cells were carefully washed once with PBS and then fixed in cold 4% paraformaldehyde for 60 s at room temperature. Fixed cells were washed in 0.05% Tween 20/PBS and stained with BCIP/NBT substrate solution in the dark for 5 to 10 min at room temperature. After one washing step with 0.05% Tween 20/PBS, PBS was added to the wells, and the optical density at 450 nm was analyzed in a microplate reader (Thermo Scientific Multiskan Spectrum). Alizarin Red staining (Sigma Aldrich, cat. no. A5533-25G) was done 21 days after the addition of osteogenic medium to human osteoblasts. Cells were washed in PBS and fixed in cold 4% paraformaldehyde for 20 min at 37 °C. Alizarin Red S solution (2%) was added for 45 min, then cells were rinsed with water, and absorption at 590 nm was measured in a microplate reader (Thermo Scientific Multiskan Spectrum).

### Ovariectomy-induced bone loss and analysis of bone density

The mouse model of ovariectomy-induced bone loss recapitulates central aspects of human postmenopausal osteoporosis (60, 61) and was performed as described (15). Briefly, 8-week-old female humanized Plexin-B1 mice were ovariectomized or sham-operated under ketamine/xylazine anesthesia. Mice were intravenously injected with anti–Plexin-B1 or IgG control antibodies (10 μg/g body weight) *via* the tail vein at days 4, 7, 14, 21, 28, 35, 42, 49, and 56 after surgery under isoflurane anesthesia. At day 63, mice were euthanized by cervical dislocation under isoflurane anesthesia, femora were collected, stored in 80% ethanol at 4 °C for at least 24 h before scanning, and subjected to microcomputed tomography (Bruker Skyscan 1276) with the following settings: X-ray source at a voltage of 70 kV, with a current 200 μA, pixel size 4 microns, rotation range 180°, rotation step 0.200 degrees, and averaging frame 7. After reconstruction (Bruker, Skyscan NRecon software), the images were morphometrically analyzed to obtain quantitative information on trabecular bone structures (Bruker, CTAn software). Reconstructed volumes were loaded into Analyze14 software (AnalyzeDirect, Mayo Clinic) for 3D segmentation of the bone structure by thresholds method. Group sizes had been designed to be equal, that is, n = 15 for both control IgG- and RbPLX7-treated mice. However, after data analysis, regenotyping of mice revealed that in the control IgG-treated group, two mice had an incorrect genotype (i.e., they were Plexin-B1–deficient mice instead of humanized Plexin-B1 mice). For this reason, these two mice had to be excluded from statistical analysis, reducing group size for IgG-treated mice to n = 13.

### Microglia isolation

Primary microglia were isolated from newborn mice (euthanized by decapitation). Brain tissue was harvested, placed into Hanks’ Balanced Salt Solution, and meninges were removed. After washing with Hanks’ Balanced Salt Solution, tissue was digested with trypsin and DNAse, cells were pelleted by centrifugation, and seeded into poly-L-lysine–coated flasks in medium containing DMEM, heat-inactivated FBS, penicillin/streptomycin, sodium pyruvate, and glutamine. Cells were incubated for 8 days at 37 °C, and the medium was changed daily for 4 days after isolation. At day 9 post isolation, cells were stimulated with 30% L-929–conditioned DMEM medium, which had been harvested after 14 days continuous cultivation of L-929 fibroblasts (obtained from ATCC). At day 14, microglia were harvested from the astrocyte layer by shaking the culture for at least 30 min at 37 °C at a frequency of 90/min. Cells were pelleted and seeded on poly-L-lysine–coated cover slips (1 × 10^5^ cells/ml). For immunostainings, primary microglia were fixed with 4% paraformaldehyde, permeabilized in 0.2% Triton/PBS, blocked in 1.5% horse serum/1%BSA/PBS, incubated with primary antibodies, washed, incubated with fluorescently labeled secondary antibodies, and analyzed using a Zeiss Ob-server Z1 AX10 fluorescence microscope. The anti-iba1 antibody was obtained from Wako (cat. no. 019-19741; dilution 1:100). The secondary antibodies were as follows: anti–human-AF488 (Jackson Immuno Research; cat. no. 109-545-098; dilution 1:200) and anti–rabbit-AF549 (Thermo Fisher cat. no. R37119; dilution 1:200).

### Experimental autoimmune encephalomyelitis

EAE in mice represents a widely accepted model of human MS (62). EAE was induced by injection of a total of 250 μg of myelin oligodendrocyte glycoprotein peptide MOG_35–55_ (Gene Script) emulsified in Complete Freund’s Adjuvant containing Incomplete Freund’s Adjuvant (BD, cat. no. 263910) and *Mycobacterium tuberculosis* H37 Ra (BD, cat. no. 231141). Injections were subcutaneous and bilateral into the flanks under isoflurane anesthesia. Pertussis toxin (Sigma Aldrich, cat. no. P7208) was injected intraperitoneally at a dose of 500 ng on the day and after 2 days of immunization with MOG_35–55_/Complete Freund’s Adjuvant. Under isoflurane anesthesia, mice were intravenously injected with anti–Plexin-B1 or IgG control antibodies (10 μg/g body weight) *via* the tail vein at days 4, 8, 11, 18, and 25 postimmunization. Mice were evaluated daily for changes in weight and clinical symptoms for 35 days using the following scoring system with observers blinded to the genotype and treatment of mice: 0 = No obvious changes in motor function; when picked up by the base of tail, the tail has tension and is erect. 0.5 = Tip of tail is limp; when picked up by the base of tail, the tail has tension except for the tip; muscle straining is felt in the tail, while the tail continues to move; intact righting reflex. 1 = Limp tail; when picked up by the base of tail, instead of being erect, the whole tail drapes over finger, no signs of tail movement are observed; intact righting reflex. 1.5 = Limp tail and hind leg inhibition; when the mouse is dropped on a wire rack, at least one hind leg falls through consistently; walking is very slightly wobbly; righting reflex delayed. 2 = Limp tail and weakness of hind legs; when the mouse is dropped on a wire rack, at least both hind legs falls through consistently; no righting reflex. 2.5 = Limp tail and dragging of hind legs; slight paralysis of the hind legs. 3 = complete paralysis of one hind leg or moderate paralysis of both hind legs. 3.5 = Limp tail and complete paralysis of hind legs. 4 = additional mild paralysis of the front legs. 4.5 = moribund. 5 = Death. Against the background that MS is three-fold more common in female than in male human patients (17, 18), mice used for EAE experiments were female. At the time of the induction of EAE, mice were 8 to 9 weeks old.

### Data and statistical analysis

Figures 2, *B*, *C*, 3, *B*, *C*, 4, *D*–*H*, S1, *A*–*E* and S2, *A*–*D* show mean values with error bars representing SDs. In Fig. S1*E*, statistical significances were evaluated by two-way ANOVA Tukey’s Post Tests. In Figure 3, *B* and *C*, statistical significances were evaluated by one-way ANOVA Tukey’s Post Tests. In Figure 4, *D*–*H*, statistical significances were evaluated by two-tailed, unpaired *t tests* using GraphPad Prism 9.0.1 software. Figures 5, *B*, *C*, and S3*E* show mean values with error bars representing SEMs and statistical significances evaluated by multiple unpaired t-tests using GraphPad Prism 9.0.1 software. ∗*p* < 0.05, ∗∗*p* < 0.01, ∗∗∗*p* < 0.001. No outliers were excluded.

## Data availability

Data are available on request from the corresponding author.

## Supporting information

This article contains supporting information.

## Conflict of interest

Certain aspects of this work are claimed within a patent application (GB2111304.8).
