# Supplementary material for: An antagonistic monoclonal anti–Plexin-B1 antibody exerts therapeutic effects in mouse models of postmenopausal osteoporosis and multiple sclerosis
Source: J Biol Chem. 2022 Jul 15;298(9):102265. doi: 10.1016/j.jbc.2022.102265 (PMC9396414; doi:10.1016/j.jbc.2022.102265)
Supplement: Supporting Information Tables [file mmc1.pdf]

## LIST OF SUPPLEMENTAL TABLES

**Table S1. Phage library selection enrichment.**

**Table S2. Binding and blocking characteristics of representative anti-Plexin-B1 clones.**

**Table S3. Inhibition of COS-7 cell collapse by RbPLX7.**

**Table S4. Comparison of key parameters between chimeric and humanized PLX7.**

**Table S5. Aggregation analysis of RbPLX7 and HuPLX7 after freeze-thaw and heat stress.**

**Table S6. List of oligos for amplification of VH and VL repertoires from cDNA.**

**Table S7. List of oligos for insertion of restriction sites and linker.**

**Table S8. List of oligos for extension overlap PCR.**

**Table S1. Phage library selection enrichment.**

| Round | Input (cfu) | Output (cfu) | | Yield (%) | | Enrichment (fold) | |
|---|---|---|---|---|---|---|---|
| | | Plexin-B1 | Strep only | Plexin-B1 | Strep only | Plexin-B1 | Strep only |
| R1 | 4.75E+11 | 3.18E+04 | 2.49E+04 | 6.69E-06 | 5.24E-06 | 1 | 1 |
| R2 | 8.20E+10 | 1.52E+06 | 5.00E+03 | 1.85E-03 | 6.10E-06 | **276.9** | 1.2 |
| R3 | 4.57E+12 | 9.07E+07 | 7.60E+05 | 1.98E-03 | 1.66E-05 | 1.1 | 2.7 |

cfu (colony forming unit)
Yield = output/input
Enrichment factor = current round yield/previous round yield

**Table S2. Binding and blocking characteristics of representative anti-Plexin-B1 clones.**

| | human Plexin-B1 binding* | cynomolgus Plexin-B1 binding* | mouse Plexin-B1 binding* | Sema4D blocking** |
|---|---|---|---|---|
| PLX5 | Yes | (Weak) | No | No |
| PLX6 | Yes | (Weak) | No | No |
| PLX7 | Yes | Yes | No | Yes |
| PLX8 | Yes | Yes | No | No |
| PLX9 | Yes | Yes | No | No |
| PLX10 | Yes | (Weak) | No | Yes |

*measured by surface plasmon resonance (SPR)
**measured by COS-7 collapse assay

**Table S3. Inhibition of COS-7 cell collapse by RbPLX7.**

| Transfection | Treatment | $EC_{50}$ (nM) |
|---|---|---|
| human Plexin-B1 | RbPLX7<br>isotype control | 0.28 ± 0.12<br>>10 |
| human Plexin-B2 | RbPLX7<br>isotype control | >150<br>>150 |
| mouse Plexin-B1 | RbPLX7<br>isotype control | >10<br>>10 |
| mouse Plexin-B2 | RbPLX7<br>isotype control | >150<br>>150 |

Each value represents the mean ±SD of two independent experiments.

**Table S4. Comparison of key parameters between chimeric and humanized PLX7.**

| | $K_D$ (nM) | Sema4D-Plexin-B1 inhibition $EC_{50}$ (nM)* | Expression yield (mg from 1l culture)** |
|---|---|---|---|
| RbPLX7 | 0.35 | 0.21 | 37.6 |
| HuPLX7 | 1.54 | 0.42 | 82.5 |

*measured by COS-7 collapse assay
**total amount purified from 1 liter expression culture

**Table S5. Aggregation analysis of RbPLX7 and HuPLX7 after freeze-thaw and heat stress.**

| Antibody | Sample | MW (kDa) | Polydispersity (Mw/Mn) | Mass fraction (%) |
|---|---|---|---|---|
| RbPLX7 | 4°C control | 143.29 (± 0.61) | 1 (± 0.01) | 98.9 |
| | RT | 146.08 (± 1.38) | 1.01 (± 0.01) | 100 |
| | 37°C | 146.84 (± 1.36) | 1.01 (± 0.01) | 100 |
| | 50°C | 166.00 (± 1.29) | 1.02 (± 0.01) | 92.1 |
| | freeze-thaw | 143.92 (± 0.93) | 1 (± 0.01) | 97.4 |
| HuPLX7 | 4°C control | 151.65 (± 2.54) | 1.03 (± 0.02) | 100 |
| | RT | 148.23 (± 1.41) | 1.01 (± 0.01) | 100 |
| | 37°C | 145.94 (± 1.25) | 1 (± 0.01) | 99.4 |
| | 50°C | 172.09 (± 2.74) | 1.03 (± 0.02) | 95.1 |
| | freeze-thaw | 146.29 (± 0.90) | 1 (± 0.01) | 98.2 |

QC criteria: Polydispersity < 1.05; Monomeric mass fraction > 95 %

**Table S6. List of oligos for amplification of VH and VL repertoires from cDNA.**

| Oligo | Sequence (5’→3’) |
|---|---|
| Rb-VH1For | CAGTCGGTGGAGGAGTCCRGG |
| Rb-VH2For | CAGTCGGTGAAGGAGTCCGAG |
| Rb-VH3For | CAGTCGYTGGAGGAGTCCGGG |
| Rb-VH4For | CAGSAGCAGCTGGWGGAGTCCGG |
| Rb-VHRev | GACTGAYGGAGCCTTAGGTTGC |
| Rb-VL1For | AGCACCGAGCTCGWKMTGACCCAGACTCCA |
| Rb-VL2For | GCCGCBCAAGTGMTGACCCAGACTGAA |
| Rb-VL3For | GCCGCBMTYGWDMTGACCCAGACTCCA |
| Rb-VL4For | GCCGAMMTYGWDMTGACCCAGACTCCA |
| Rb-VL1Rev | TTTGACGACCACCTCGGTCCC |
| Rb-VL2Rev | TAGGATCTCCAGCTCGGTCCC |
| Rb-VL3Rev | TTTGAYTTCCACMTTGGTSCC |

**Table S7. List of oligos for insertion of restriction sites and linker.**

| Oligo | Sequence (5’→3’) |
|---|---|
| Rb-SfiI-VH1For | GTTCCTTTCTATGCGGCCCAGCCGGCCATGGCCCAGTCGCTGCAGGA GTCCRRG |
| Rb-SfiI-VH2For | GTTCCTTTCTATGCGGCCCAGCCGGCCATGGCCCAGSAGCAGCTGCA GGAGTCCGG |
| Rb-VH-linkRev | CCTTCAGAAGACTTCCCAGAACCAGAAGTAGAACCSGAGGAGACGGT GACCAGGGTSCCYKKGCCCCA |
| Rb-Link-VL1For | CTTCTGGTTCTGGGAAGTCTTCTGAAGGAAAAGGCGACATYGAGCTC ACCCAGACTCCA |
| Rb-Link-VL2For | CTTCTGGTTCTGGGAAGTCTTCTGAAGGAAAAGGCGACCAAGAGCTC ACCCAGACTGAA |
| Rb-VL1-NotI-Rev | GAATCGTCATCTGCGGCCGCTTTGAYTTCCACMTTGGTSCC |
| Rb-VL2-NotI-Rev | GAATCGTCATCTGCGGCCGCTWKGAYSWCCASCTCGGTCCC |

**Table S8. List of oligos for extension overlap PCR.**

| Oligo | Sequence (5'→3') |
|---|---|
| Rb-PTH-For | GTTCCTTTCTATGCGGCCCAGCCGGCCATGGCC |
| Rb-PTL-Not-Rev | GAATCGTCATCTGCGGCCGC |
