# Supplementary material for: An antagonistic monoclonal anti–Plexin-B1 antibody exerts therapeutic effects in mouse models of postmenopausal osteoporosis and multiple sclerosis
Source: J Biol Chem. 2022 Jul 15;298(9):102265. doi: 10.1016/j.jbc.2022.102265 (PMC9396414; doi:10.1016/j.jbc.2022.102265)
Supplement: Supporting Information Figure Legends [file mmc2.pdf]

Figure S1. Serum screening of rabbits immunized with human Plexin-B1 (20-535). ELISA binding assays of immune sera samples collected on day 0, 21 and 35 after immunization. *A and B*, Rabbit 88 (Rb88) serum against (A) human Plexin-B1 and (B) streptavidin control. *C and D*, Rabbit 89 (Rb89) serum against (C) human Plexin-B1 and (D) streptavidin control. Data points in A-D were fitted using four-parameters variable slope non-linear regression fit (GraphPad Prism). *E*, Competition ELISA assay using human, cynomolgus and mouse orthologs of Plexin-B1 to assess cross-reactivity. Immune sera were collected on day 21 after immunization, and pre-incubated with excess of human, cynomolgus and mouse Plexin-B1 (20-535) proteins, before testing binding to plates with biotinylated human Plexin-B1 (20-535). A drop of the ELISA signal indicates binding and depletion of anti-Plexin-B1 reactive antibodies during the pre-incubation step. All graphs show mean values ± s.d..

Figure S2. Sema4D dose response in the COS-7 collapse assay. *A-D*, COS-7 cells overexpressing (A) human Plexin-B1, (B) mouse Plexin-B1, (C) human Plexin-B2, or (D) mouse Plexin-B2 were incubated with the indicated concentrations of Sema4D, followed by image analysis to assess the morphology of individual wells. The number of collapsed cells per well was quantified manually. EC_{50} values were calculated using five-parameters non-linear regression analysis in GraphPad Prism. Shown are representative examples of 2 independent experiments (biological replicates). Graphs depict mean values ± s.d..

Figure S3. Generation and validation of a humanized Plexin-B1 mouse line. *A* and *B*, RNA was isolated from the femora of mice carrying one functional *plxnb1* allele (*plxnb1*$^{+/-}$; mice carry one wildtype allele and one knockout allele), from the femora of Plexin-B1 knockout mice (*plxnb1*$^{-/-}$), or from the femora of “humanized Plexin-B1 mice” (“Plexin-B1 humanized”) expressing the human *plxnb1* gene instead of the endogenous murine *plxnb1* gene. Shown are RT-PCRs, which specifically detect the (A) murine *plxnb1* mRNA (but not the human *plxnb1* mRNA) and (B) human *plxnb1* mRNA (but not the murine *plxnb1* mRNA). *C*, RNA was isolated from microglia isolated from mice with the indicated genotypes. Shown is an RT-PCR, which

specifically detects the human *plxnb1* mRNA (but not the murine *plxnb1* mRNA). Each lane represents microglia from an individual mouse. *D*, Microglia isolated from a Plexin-B1 knockout mouse and from a humanized Plexin-B1 mouse were immunostained for the microglia marker iba1 (red) and for Plexin-B1 (antibody RbPLX7; green). Scale bar, 40 µm. *E*, Humanized Plexin-B1 mice ("Plexin-B1 humanized") and Plexin-B1 knockout mice were subjected to EAE, and clinical score was analyzed. Shown are mean values ± s.e.m.; humanized Plexin-B1 mice: n=7; *plxnb1*$^{-/-}$ mice: n=7.
