# Supplementary figures and images for: An antagonistic monoclonal anti–Plexin-B1 antibody exerts therapeutic effects in mouse models of postmenopausal osteoporosis and multiple sclerosis
Source: J Biol Chem. 2022 Jul 15;298(9):102265. doi: 10.1016/j.jbc.2022.102265 (PMC9396414; doi:10.1016/j.jbc.2022.102265)

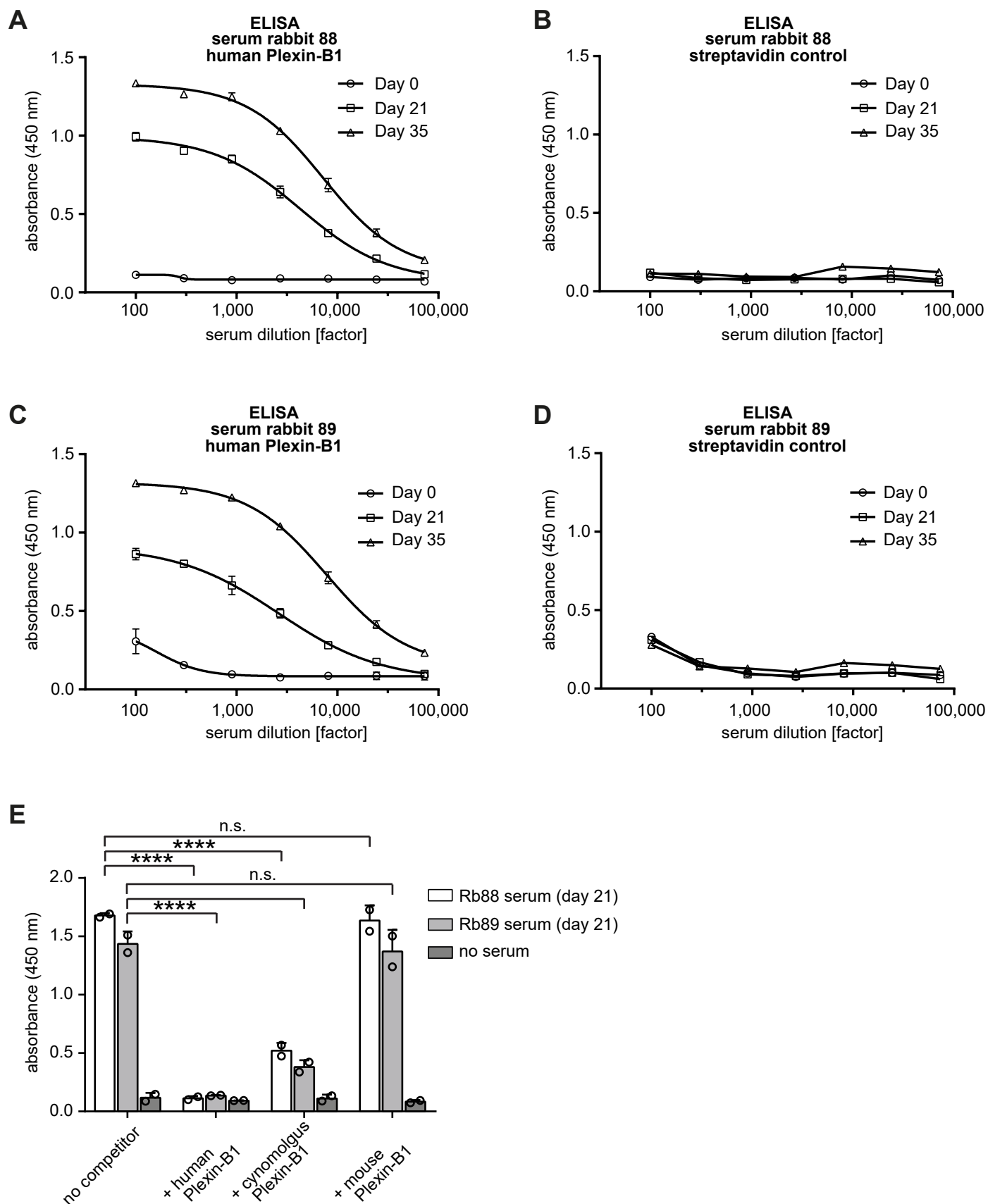

Figure S1

Supplement: Figure S1 [file mmc3.pdf]

**A**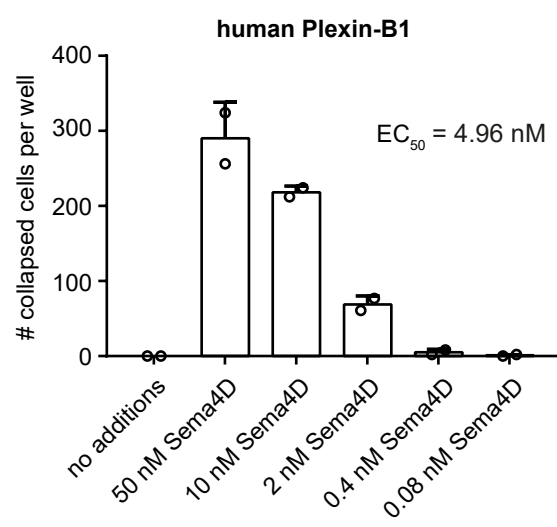**B**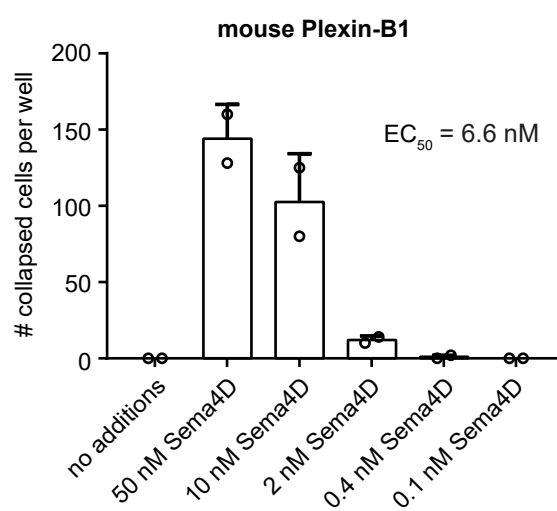**C**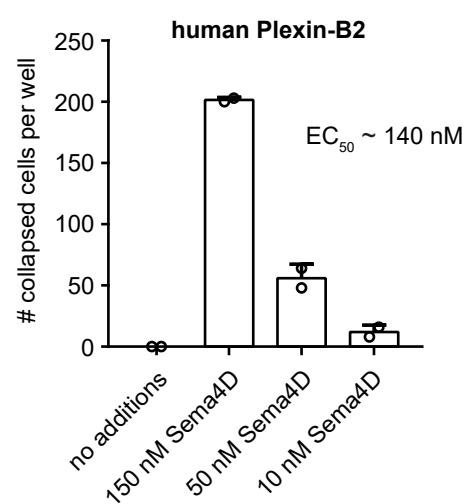**D**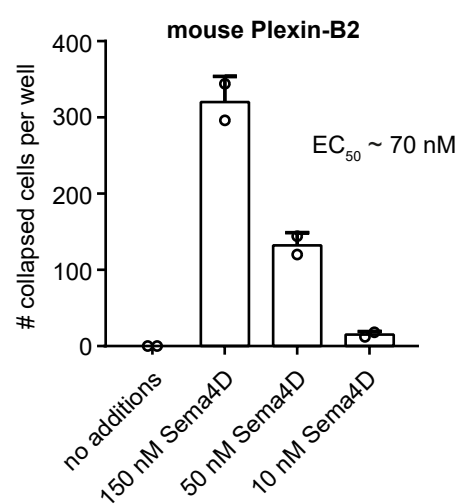

Figure S2

Supplement: Figure S2 [file mmc4.pdf]

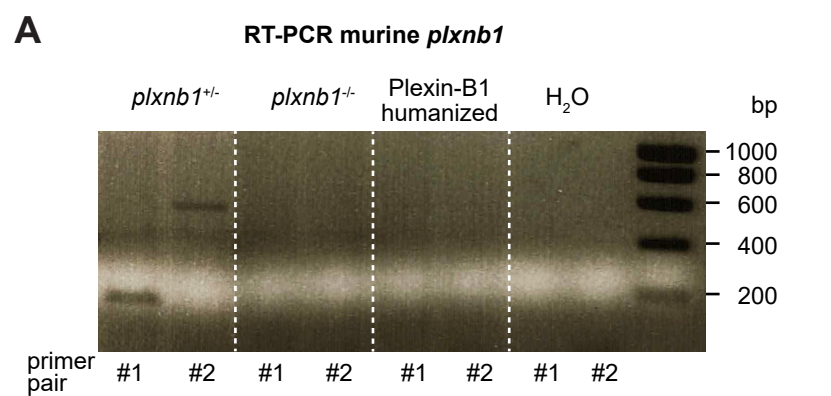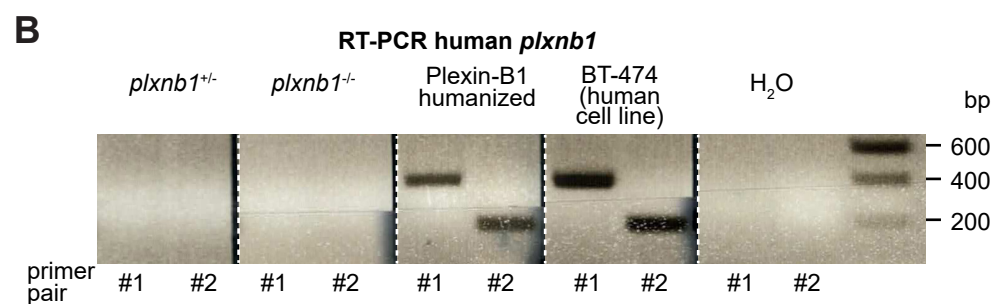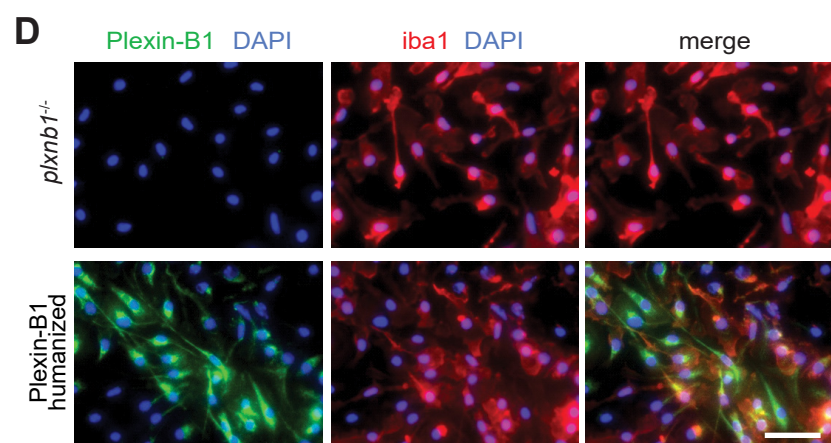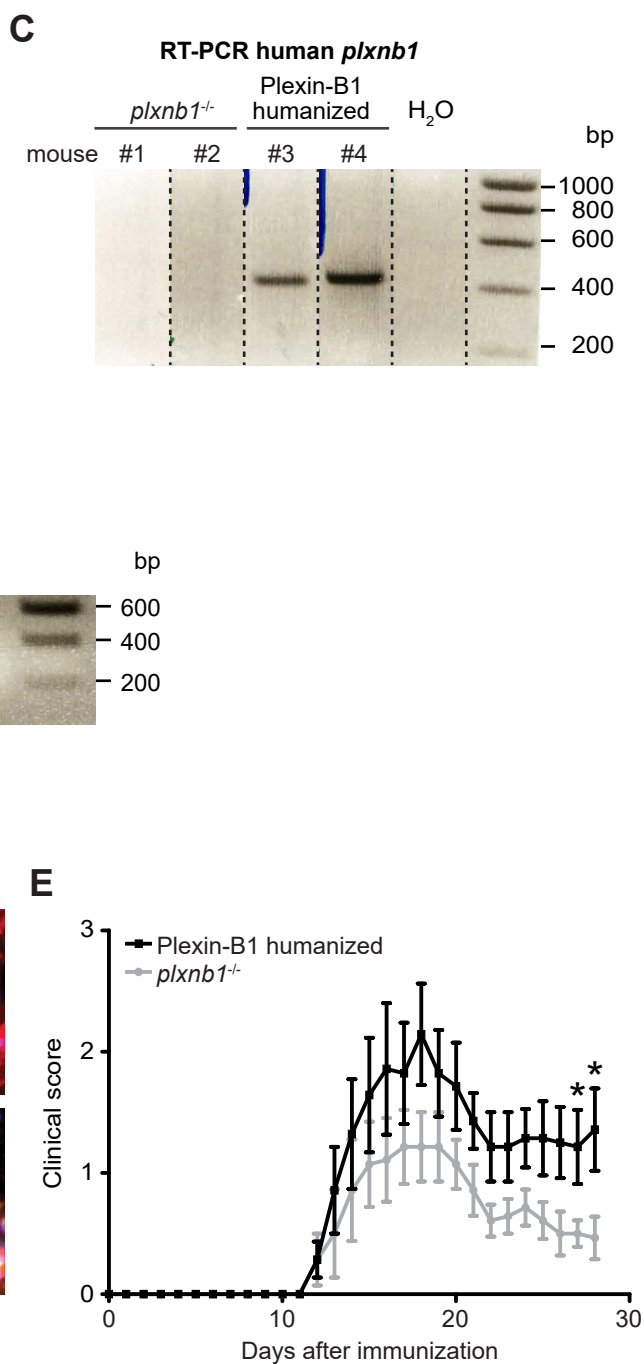

Figure S3

Supplement: Figure S3 [file mmc5.pdf]
